# Supplementary material for: Distinct Postprandial Bile Acids Responses to a High-Calorie Diet in Men Volunteers Underscore Metabolically Healthy and Unhealthy Phenotypes
Source: Nutrients. 2020 Nov 19;12(11):3545. doi: 10.3390/nu12113545 (PMC7699492; doi:10.3390/nu12113545)
Supplement: Supplementary file 1 [file nutrients-12-03545-s001.pdf]

Supplemental Table S1: Bile acid molecular species.

Component names	Bile acid types	Acronyms	m/z	MRM	CE (V)	RT (min)
3α,7α-dihydroxy-5β-cholanic acid	I	CDCA	391.4	391.4>391.4	-10	15.16
3α,7α,12α-trihydroxy-5β-cholanic acid	I	CA	407.4	407.4>407.4	-10	12.22
3α,12α-dihydroxy-5β-cholanic acid	II	DCA	391.4	391.4>391.4	-10	15.46
3α-5β-cholanic acid	II	LCA	375.4	375.4>375.4	-10	17.58
3α,7b-dihydroxy-5β-cholanic acid	II	UDCA	391.4	391.4>391.4	-10	11.30
3α,6α,17α-trihydroxy-5β-cholanic acid	I	HCA	407.4	407.4>407.4	-10	10.92
3α,6α-dihydroxy-5β-cholanic acid	II	HDCA	391.4	391.4>391.4	-10	12.19
3α,6β,7β-trihydroxy-5β-cholanic acid	I	βMCA	407.4	407.4>407.4	-10	9.00
Glyco 3α,7α-dihydroxy-5β-cholanic acid	I	GCDCA	448.4	448.4>74.0	-70	12.01
Glyco 3α,7α,12α-trihydroxy-5β-cholanic acid	I	GCA	464.4	464.4>74.0	-70	8.45
Glyco 3α,12α-dihydroxy-5β-cholanic acid	II	GDCA	448.4	448.4>74.0	-70	12.65
Glyco 3α-5β-cholanic acid	II	GLCA	432.4	432.4>74.0	-70	15.00
Glyco 3α,7β-dihydroxy-5β-cholanic acid	II	GUDCA	448.4	448.4>74.0	-70	7.08
Tauro 3α,7α-dihydroxy-5β-cholanic acid	I	TCDCA	498.4	498.4>80.0	-110	12.07
Tauro 3α,7α,12α-trihydroxy-5β-cholanic acid	I	TCA	514.4	514.4>80.0	-110	8.74
Tauro 3α,12α-dihydroxy-5β-cholanic acid	II	TDCA	498.4	498.4>80.0	-110	12.65
Tauro 3α-5β-cholanic acid	II	TLCA	482.4	482.4>80.0	-110	14.90
Tauro 3α,7β-dihydroxy-5β-cholanic acid	II	TUDCA	498.4	498.4>80.0	-110	7.45
Tauro 3α,6α-dihydroxy-5β-cholanic acid	II	THDCA	498.4	498.4>80.0	-110	8.10
3α,7α-dihydroxy-5β-cholanic acid 3Sulfate	I	CDCA3S	471.4	471.4>97.0	-70	11.01
3α,7α,12α-trihydroxy-5β-cholanic acid 3Sulfate	I	CA3S	497.4	497.4>97.0	-70	7.75
3α,12α-dihydroxy-5β-cholanic acid 3Sulfate	II	DCA3S	471.4	471.4>97.0	-70	8.18
3α-5β-cholanic acid 3Sulfate	II	LCA3S	455.4	455.4>97.0	-70	13.62
3α,7β-dihydroxy-5β-cholanic acid 3Sulfate	II	UDCA3S	471.4	471.4>97.0	-70	7.04
Glyco 3α-5β-cholanic acid	II	GLCA3S	512.4	512.4>97.0	-70	9.20
Glyco 3α,7β-dihydroxy-5β-cholanic acid 3Sulfate	II	GUDCA3S	528.4	528.4>97.0	-70	3.75
Tauro 3α-5β-cholanic acid 3 Sulfate	II	TLCA3S	280.8	280.8>97.0	-70	9.25
Tauro 3α,7β-dihydroxy-5β-cholanic acid 3Sulfate	II	TUDCA3S	288.9	288.9>97.0	-70	3.91
was 23-nor-5β-cholanoic acid-3α,12α diol	IS	Nor	377.4	377.4>377.4	-10	13.29

Type: I primary, II secondary and IS (Internal Standard) . MRM (Multiple Reaction Monitoring) quantifier MRM. CE (collision energy). RT (Retention Time).

Supplemental Table S2: C4 quantification.

Component names	Acronyms	m/z	MRM	MRM Type	CE (V)	RT(min)
**7α-hydroxy-4-cholesten-3-one**	C4	401.3	401.3>97.0	quantifier	30	7.34
**7α-hydroxy-4-cholesten-3-one**	C4	401.3	401.3>177.2	qualifier	25	7.34
**D7, 7α-hydroxy-4-cholesten-3-one**	D7-C4	408.3	408.3>97.0	quantifier	30	7.29
**D7, 7α-hydroxy-4-cholesten-3-one**	D7-C4	408.3	408.3>177.2	qualifier	25	7.29

MRM (Multiple Reaction Monitoring) quantifier MRM. CE (collision energy). RT (Retention Time).

Supplemental Table S3. Limit of detection and quantification for each analyte.

Analyte name	acronym	LOQ (nM)	LOD (nM)
3α,7α-dihydroxy-5β-cholanic acid	CDCA	0.82	0.1
3α,7α,12α-trihydroxy-5β-cholanic acid	CA	0.85	0.2
3α,12α-dihydroxy-5β-cholanic acid	DCA	0.93	0.1
3α-5β-cholanic acid	LCA	0.95	0.3
3α,7b-dihydroxy-5β-cholanic acid	UDCA	0.92	0.1
3α,6α,17α-trihydroxy-5β-cholanic acid	HCA	0.86	0.3
3α,6α-dihydroxy-5β-cholanic acid	HDCA	0.87	0.2
3α,6b,7b-trihydroxy-5β-cholanic acid	bMCA	0.78	0.1
Glyco 3α,7α-dihydroxy-5β-cholanic acid	GCDCA	0.8	0.2
Glyco 3α,7α,12α-trihydroxy-5β-cholanic acid	GCA	0.75	0.3
Glyco 3α,12α-dihydroxy-5β-cholanic acid	GDCA	0.83	0.3
Glyco 3α-5β-cholanic acid	GLCA	0.8	0.4
Glyco 3α,7b-dihydroxy-5β-cholanic acid	GUDCA	0.83	0.3
Tauro 3α,7α-dihydroxy-5β-cholanic acid	TCDCA	0.78	0.2
Tauro 3α,7α,12α-trihydroxy-5β-cholanic acid	TCA	0.71	0.3
Tauro 3α,12α-dihydroxy-5β-cholanic acid	TDCA	0.79	0.2
Tauro 3α-5β-cholanic acid	TLCA	0.65	0.1
Tauro 3α,7b-dihydroxy-5β-cholanic acid	TUDCA	1.5	0.5
Tauro 3α,6α-dihydroxy-5β-cholanic acid	THDCA	0.66	0.2
3α,7α-dihydroxy-5β-cholanic acid 3Sulfate	CDCA3S	0.83	0.3
3α,7α,12α-trihydroxy-5β-cholanic acid 3Sulfate	CA3S	0.75	0.4
3α,12α-dihydroxy-5β-cholanic acid 3Sulfate	DCA3S	0.8	0.2
3α-5β-cholanic acid 3Sulfate	LCA3S	0.8	0.3
3α,7b-dihydroxy-5β-cholanic acid 3Sulfate	UDCA3S	0.77	0.2
Glyco 3α-5β-cholanic acid	GLCA3S	0.72	0.1
Glyco 3α,7b-dihydroxy-5β-cholanic acid 3Sulfate	GUDCA3S	0.61	0.3
Tauro 3α-5β-cholanic acid 3 Sulfate	TLCA3S	0.87	0.1
Tauro 3α,7b-dihydroxy-5β-cholanic acid 3Sulfate	TUDCA3S	0.62	0.2
23-nor-5b-cholanoic acid-3a,12a diol	Nor	0.65	0.1
7α-hydroxy-4-cholesten-3-one	C4	0.49	0.24

LOD: Limit of detection; LOQ: Limit of quantification.

Supplemental Table S4. Relationship between BAs levels and clinical and biological parameters.

	Total BAs	Primary BAs	Secondary BAs	Tertiary BAs	Sulfo-Conj BAs	Unconj BAs	Conj BAs	C4
Age	**-0.255**	**-0.261**	-0.081	**-0.278**	-0.059	-0.172	**-0.300**	-0.078
BMI	-0.191	-0.140	-0.114	-0.143	-0.083	<u>-0.201</u>	-0.181	0.040
Waist	<u>-0.202</u>	-0.189	-0.033	0.039	0.060	**-0.240**	-0.091	0.007
Total Cholesterol	**-0.384**	**-0.301**	**-0.331**	<u>-0.216</u>	-0.050	**-0.414**	**-0.238**	-0.064
LDL-C	**-0.325**	-0.226	**-0.360**	<u>-0.226</u>	-0.090	**-0.367**	<u>-0.225</u>	-0.179
HDL-C	-0.103	-0.109	0.071	0.001	0.124	-0.072	-0.087	0.064
FBG	-0.084	-0.108	-0.058	-0.033	0.016	-0.121	-0.071	0.043
Hba1c	-0.194	-0.176	**-0.255**	-0.054	-0.048	<u>-0.232</u>	0.003	-0.059
Insulin	0.110	0.085	0.085	0.199	0.091	0.015	0.192	0.095
HOMA-IR	0.111	0.084	0.071	0.178	0.081	0.017	0.181	0.106

BMI: Body mass index; FBG: fasting blood glucose; significant correlation are highlighted in bold face while trends ($p<0.1$) are underlined.

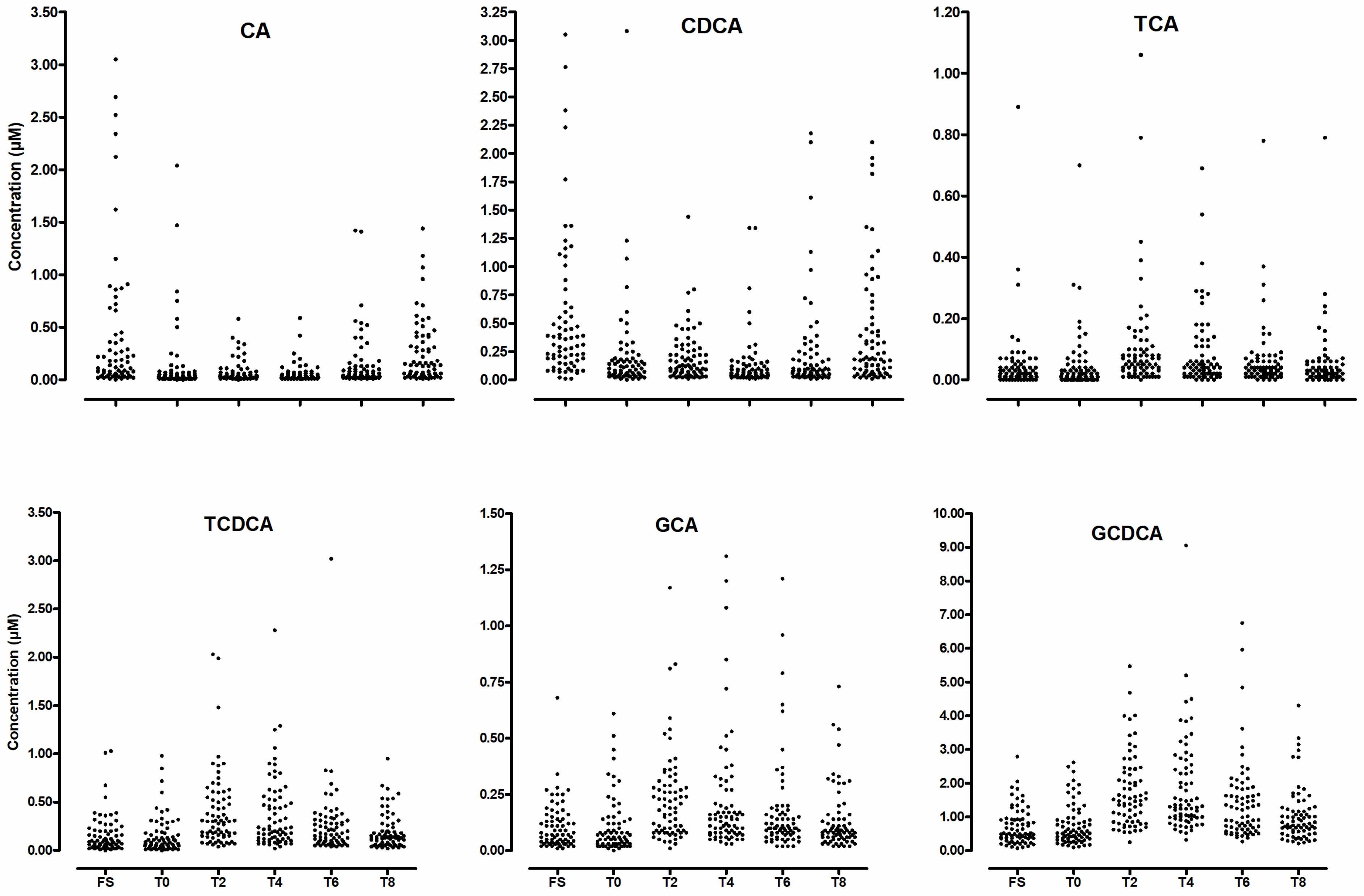

Figure S1: Interindividual variability of circulating levels of primary BA species during postprandial exploration determined in 71 healthy subjects : FS for overnight fasting samples, T0, T2, T4, T6, T8 for before and 2h, 4h, 6h and 8h after consumption of a hypercaloric high fat test meal.

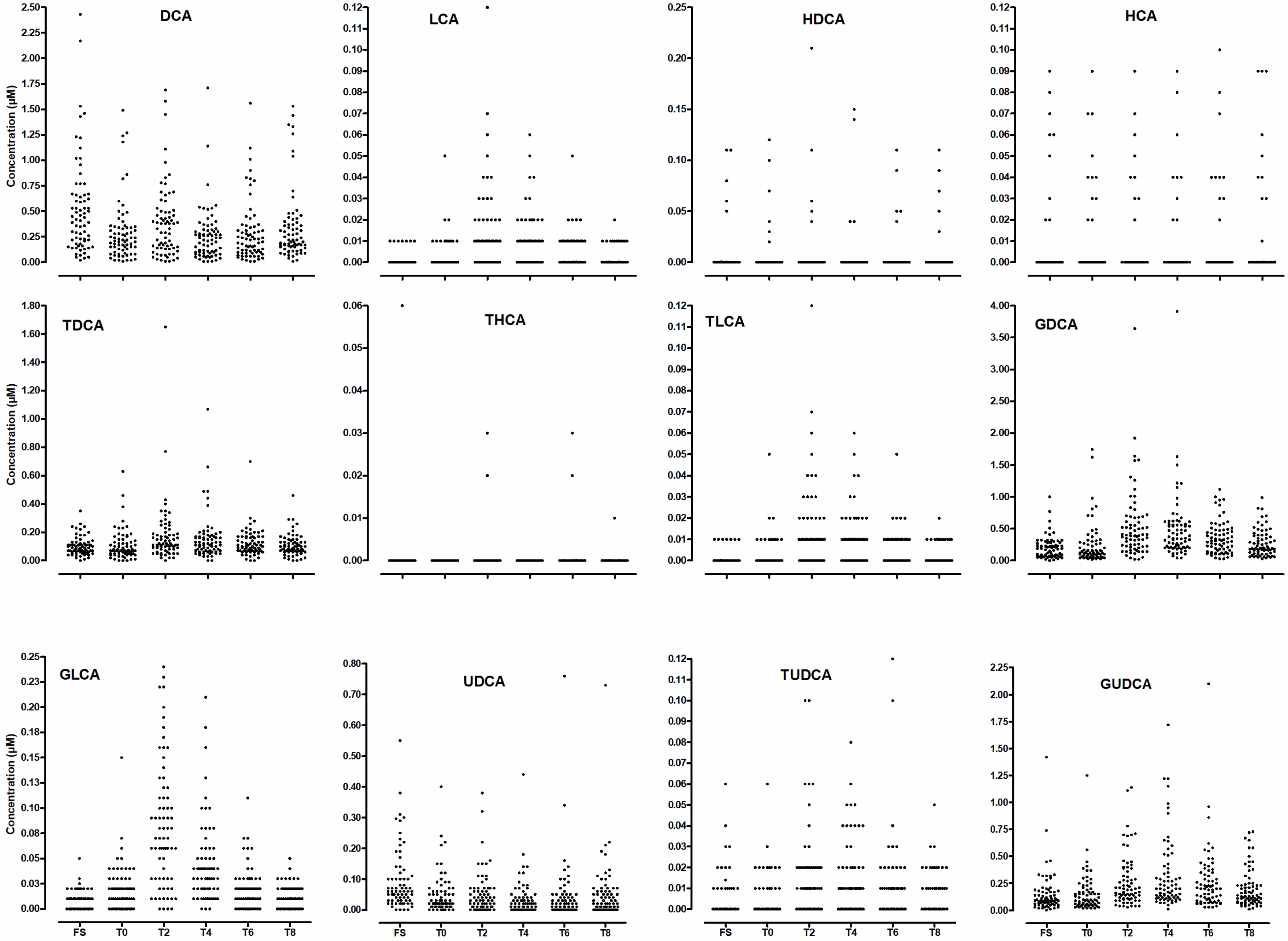

Figure S2: Interindividual variability of circulating levels of secondary and tertiary BA species during postprandial exploration determined in 71 healthy subjects: FS for overnight fasting samples, T0, T2, T4, T6, T8 for before and 2h, 4h, 6h and 8h after consumption of a hypercaloric high fat test meal.

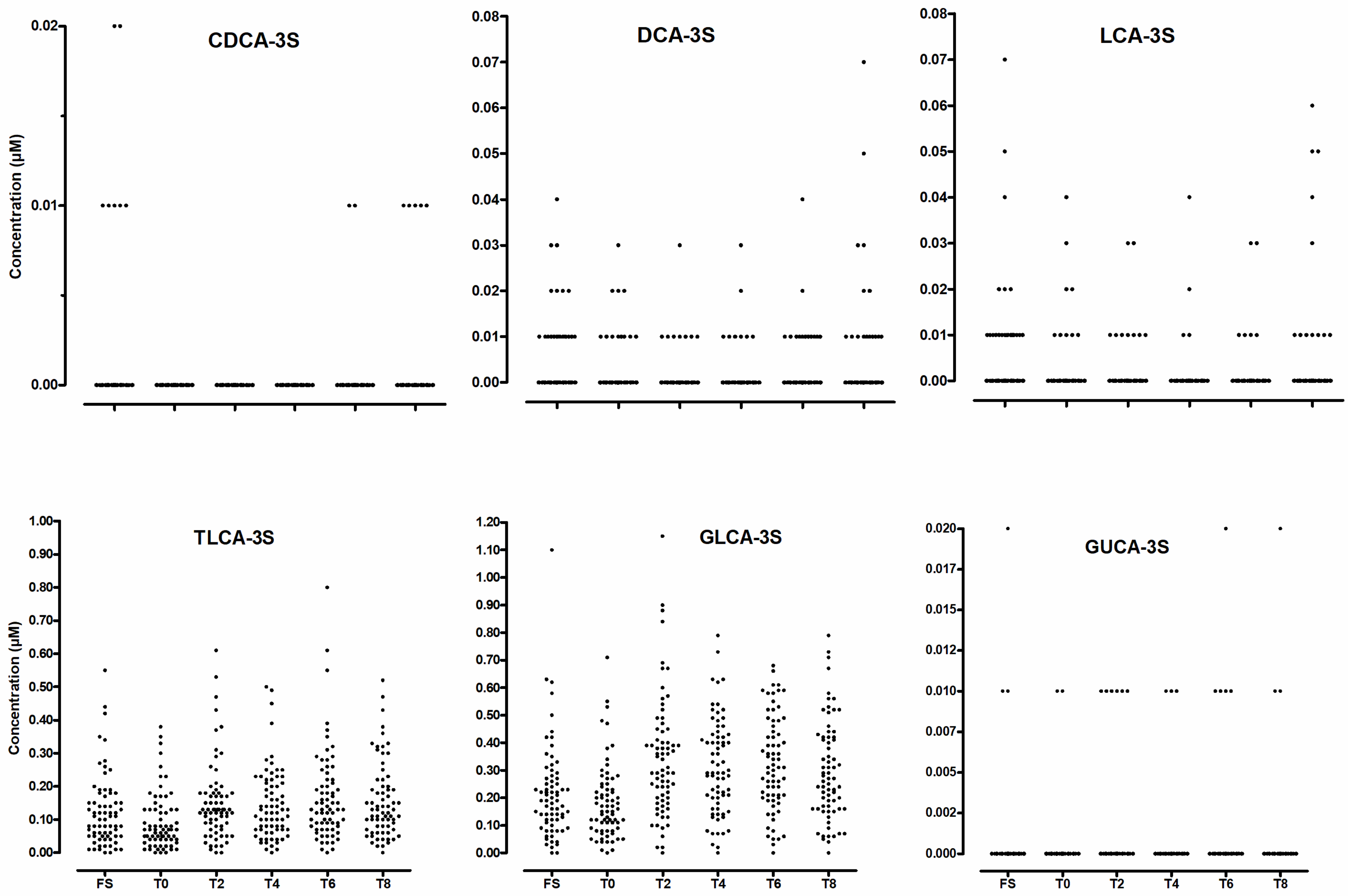

Figure S3: Interindividual variability of circulating levels of sulfated BA species during postprandial exploration determined in 71 healthy subjects: FS for overnight fasting samples, T0, T2, T4, T6, T8 for before and 2h, 4h, 6h and 8h after consumption of a hypercaloric high fat test meal.

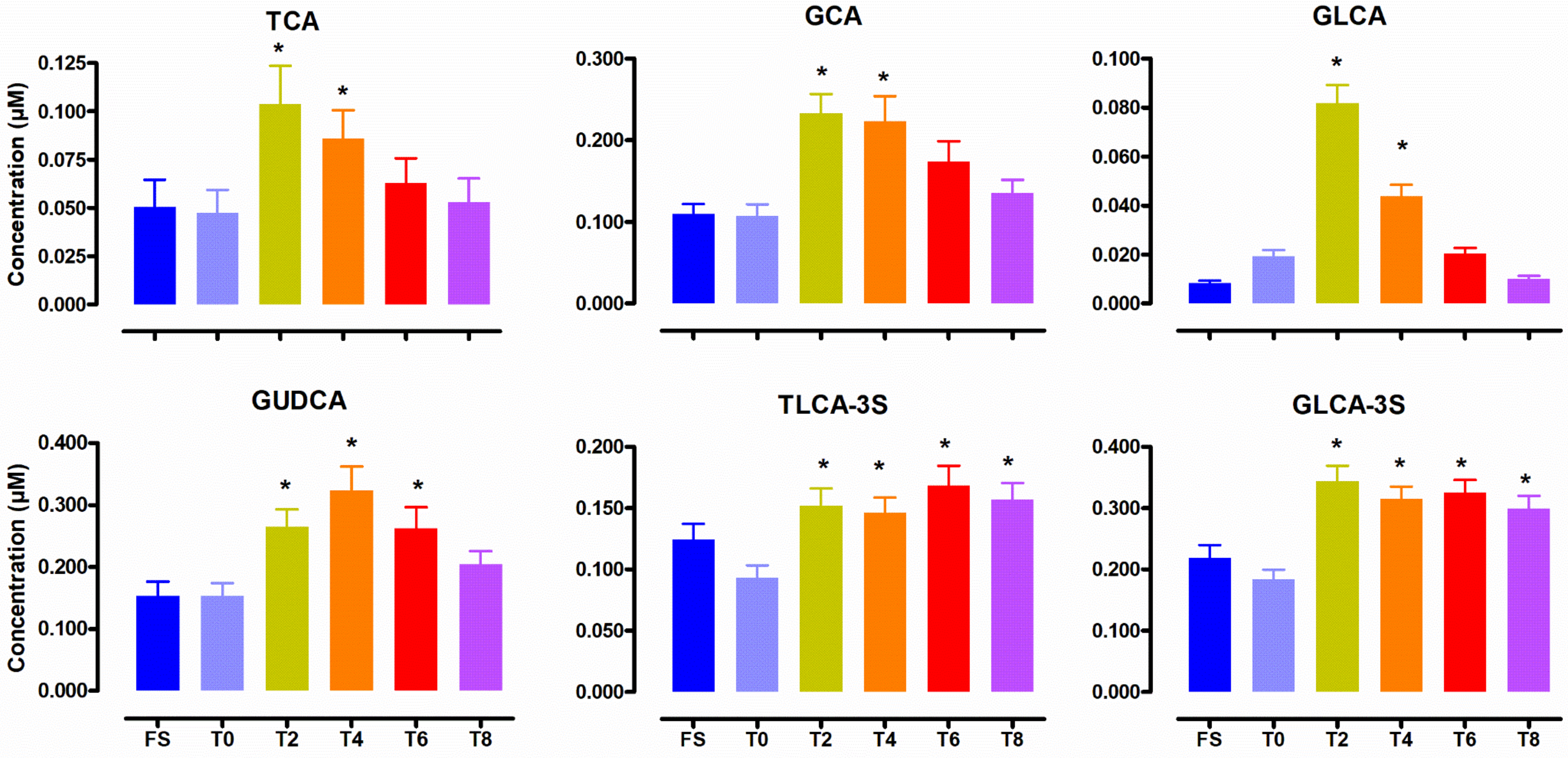

Figure S4: Mean circulating levels of individual species of conjugated BAs during postprandial exploration: FS for overnight fasting samples, T0, T2, T4, T6, T8 for before and 2h, 4h, 6h and 8h after consumption of a hypercaloric high fat test meal. Values are mean ± SEM.

* $p<0.05$ versus overnight fasting sample.

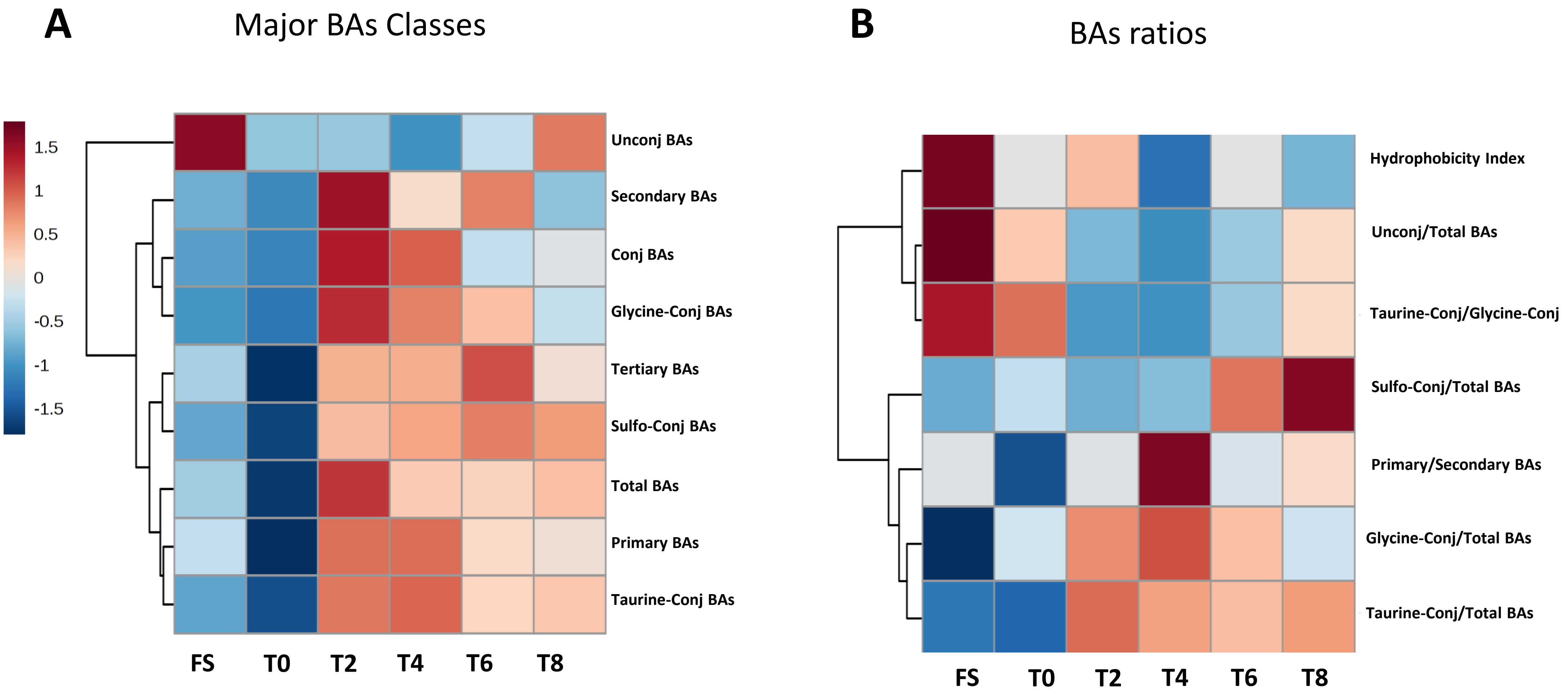

Figure S5: Hierarchical cluster analysis of qualitative features of plasma BAs according to postprandial time course, FS for overnight fasting samples, T0, T2, T4, T6, T8 for before and 2h, 4h, 6h and 8h after consumption of a hypercaloric high fat test meal.

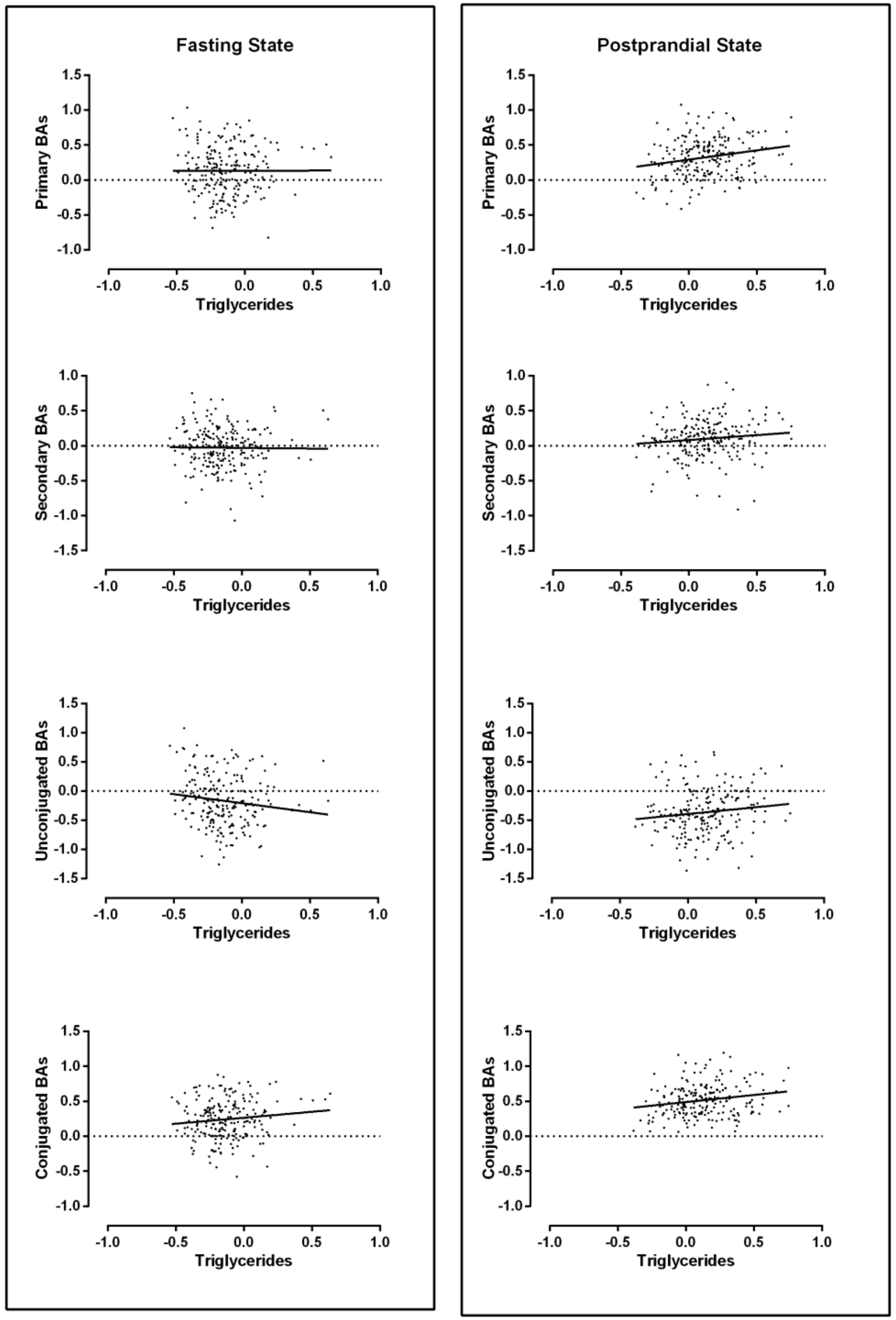

Figure S6: Scatter plots showing relationship between log-transformed circulating levels of major class of BA (Primary BAs, Secondary BAs, Unconjugated BAs and Conjugated BAs) and triglycerides in both fasting and postprandial states**,** Samples collected after an overnight fast (FS), before (T0) and after 8 hours (T8) after test meal intake were used to assess the relationship between bile acids and triglycerides levels in fasting state (n=213). Samples collected 2 hours (T2), 4 hours (T4) and 6 hours (T6) after test meal intake was used to assess the relationship between bile acids and triglycerides levels in postprandial state (n=213).

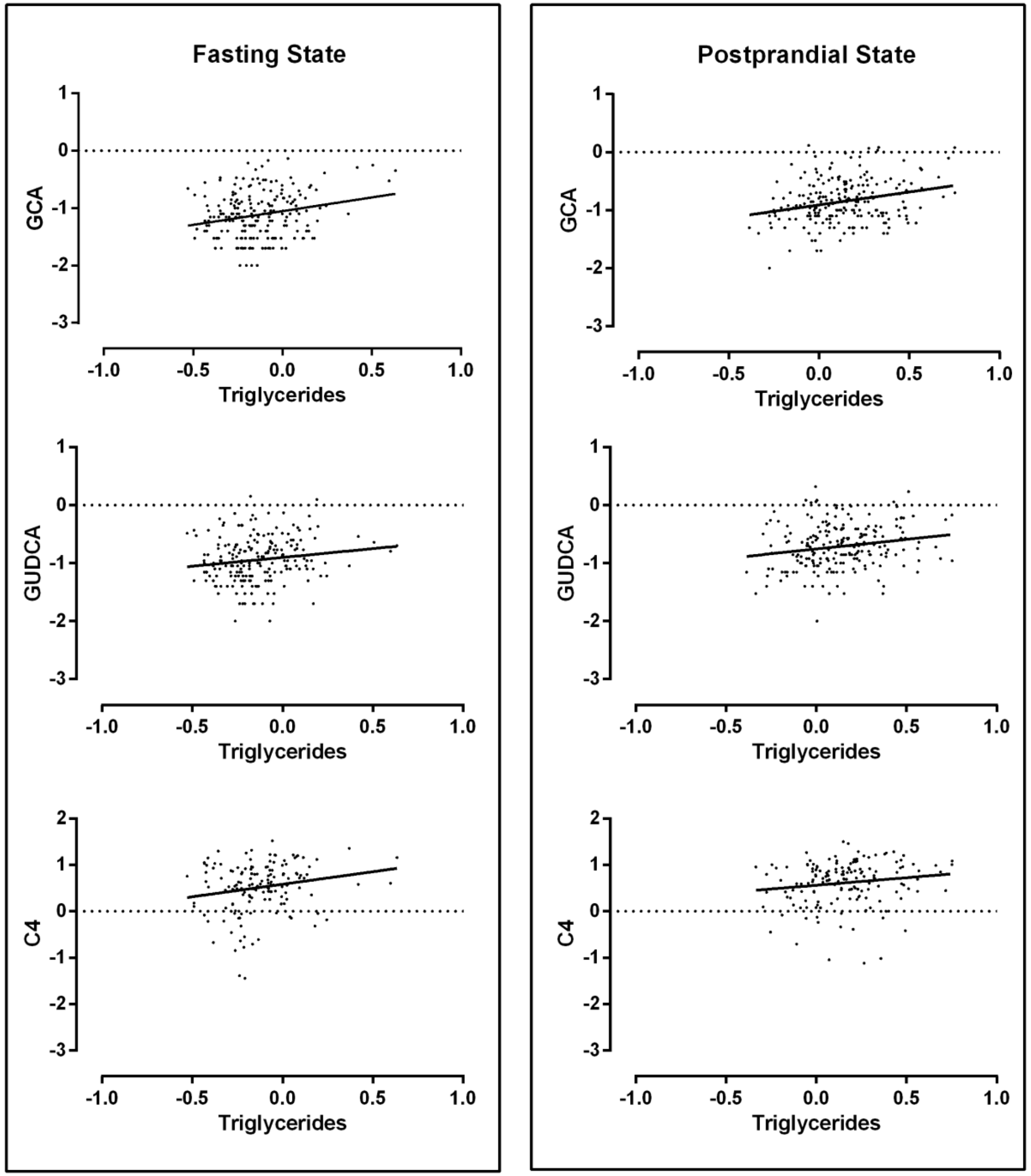

Figure S7: Scatter plots showing relationship between log-transformed circulating levels GCA, GUDCA or C4 and triglycerides in both fasting and postprandial states**.** Samples collected after an overnight fast (FS), before (T0) and after 8 hours (T8) after test meal intake were used to assess the relationship between bile acids and triglycerides levels in fasting state (n=213). Samples collected 2 hours (T2), 4 hours (T4) and 6 hours (T6) after test meal intake was used to assess the relationship between bile acids and triglycerides levels in postprandial state (n=213).

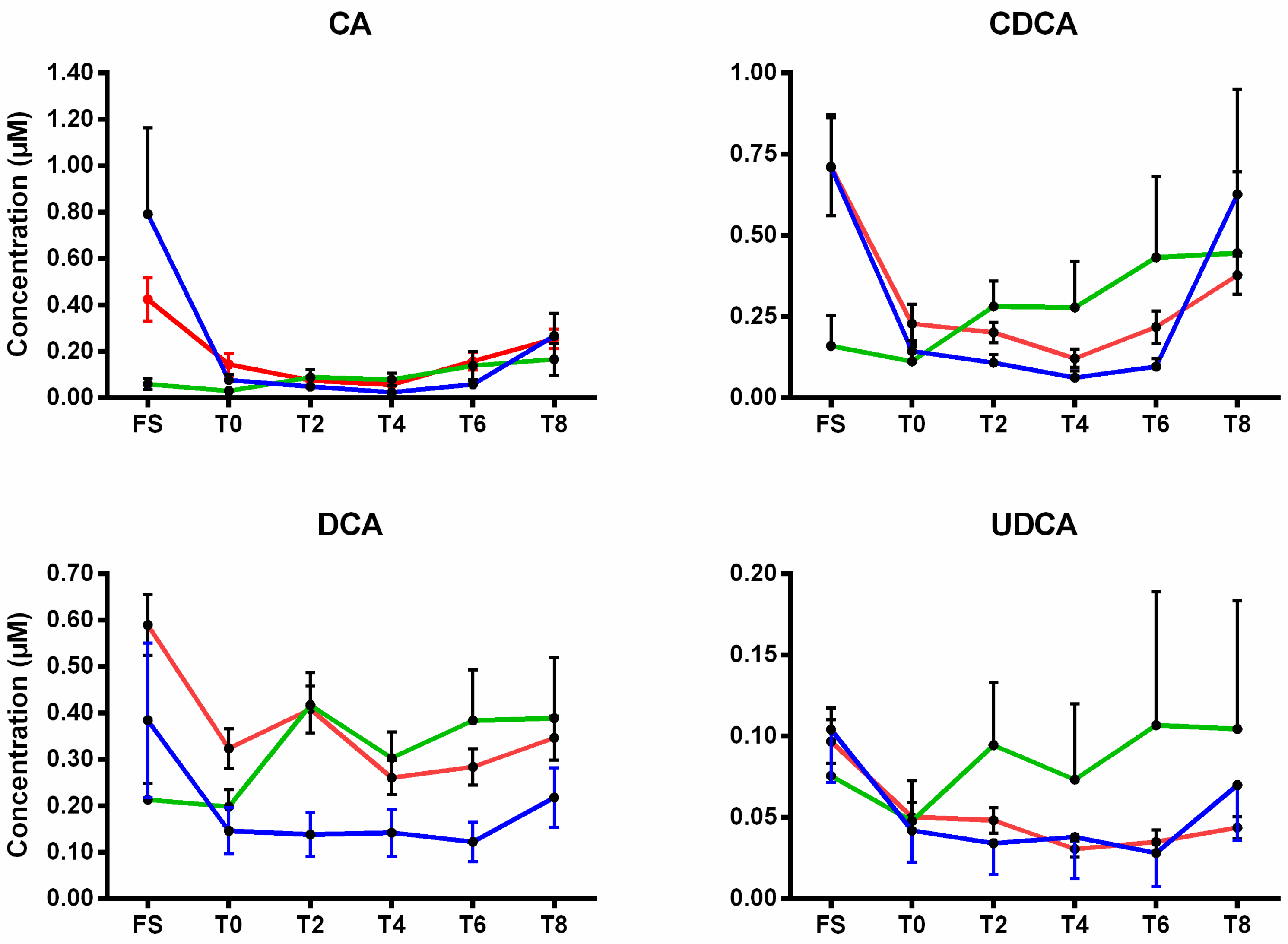

Figure S8: Circulating levels of unconjugated BA species during postprandial time course in subjects from cluster C0 (red line), C1 (green line) and C2 (blue line). Values are mean ± SEM.

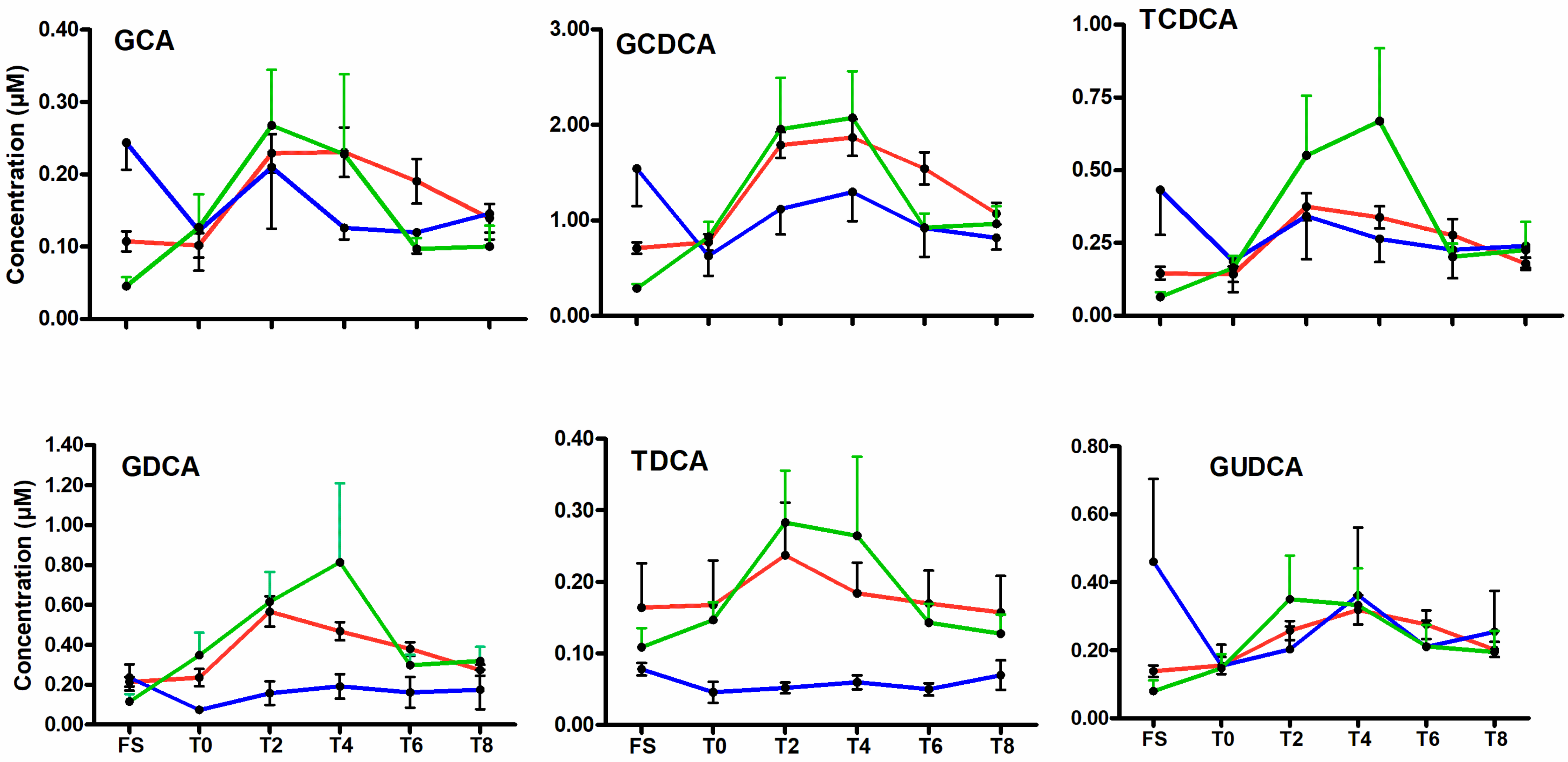

Figure S9: Circulating levels of conjugated BA species during postprandial time course in subjects from cluster C0 (red line), C1 (green line) and C2 (blue line). Values are mean ± SEM.

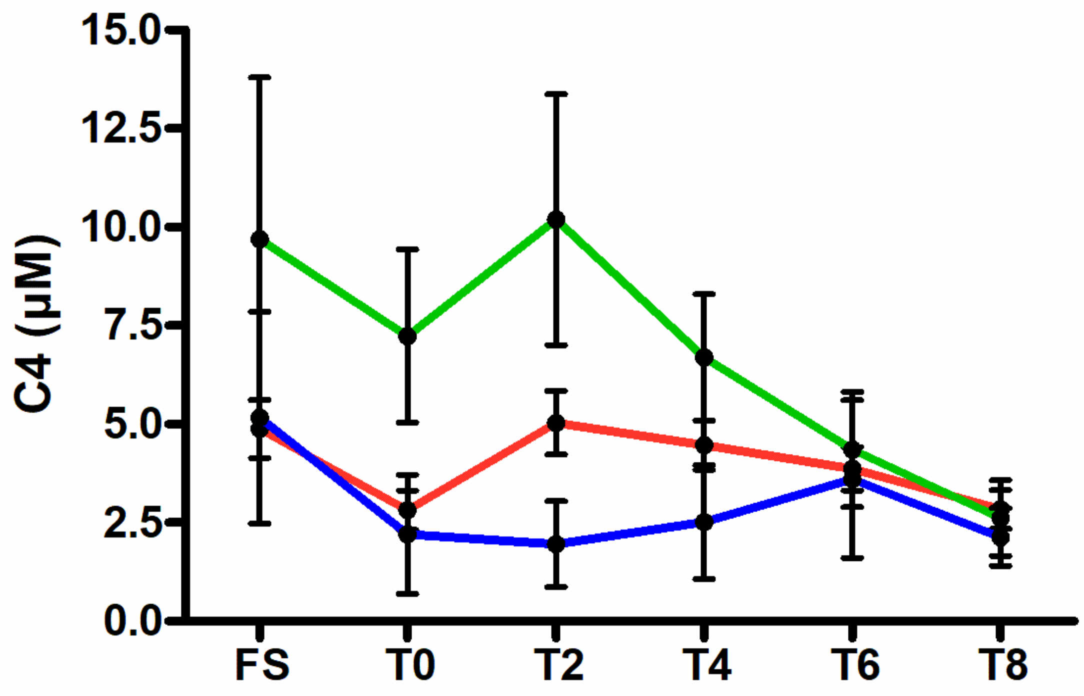

Figure S10: Circulating levels of BA synthesis marker 7α-hydroxy-4-cholesten-3-one, C4, during postprandial time course in subjects from cluster C0 (red line), C1 (green line) and C2 (blue line). Values are mean ± SEM.

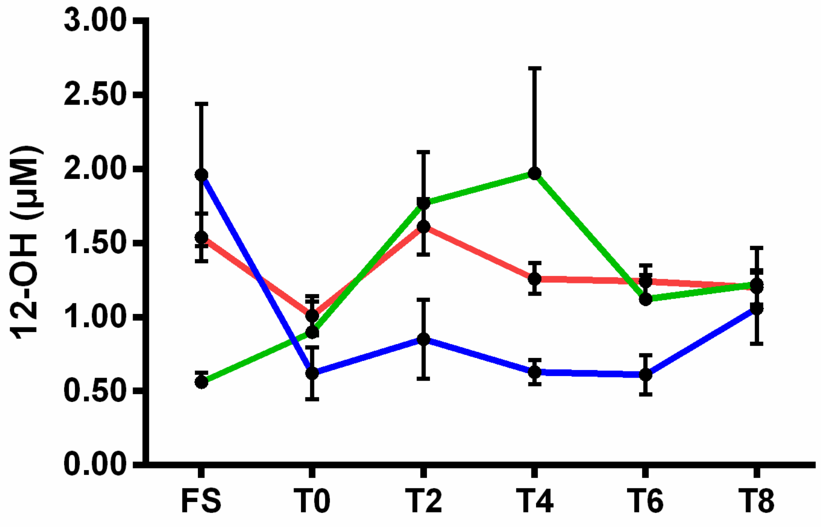

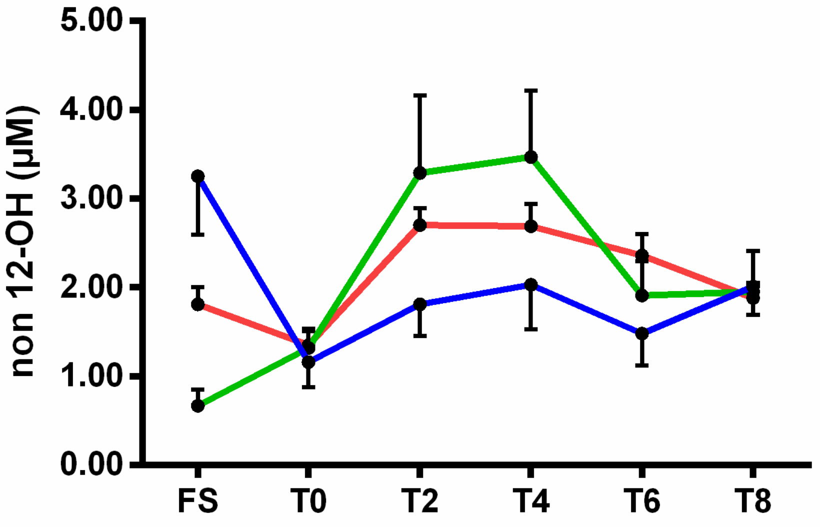

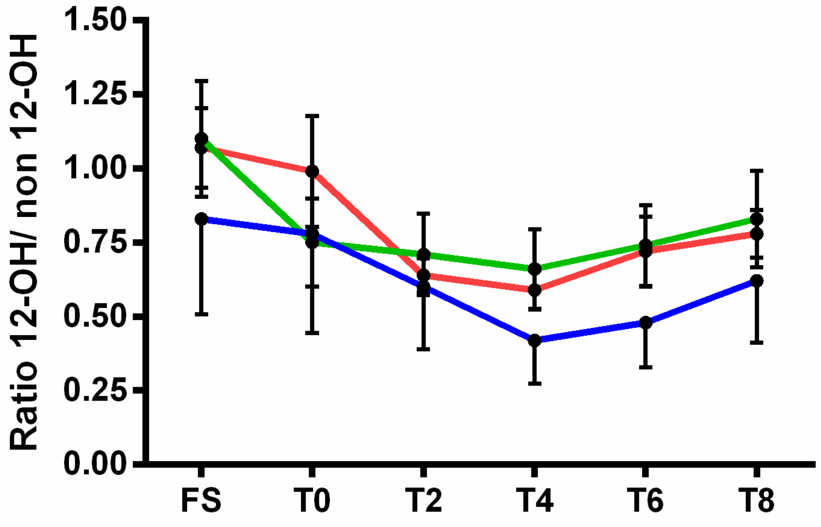

Figure S11: Circulating levels of 12-OH and non 12-OH BAs and variation of 12-OH to non 12-OH BAs ratio during postprandial time course in subjects from cluster C0 (red line), C1 (green line) and C2 (blue line). Values are mean ± SEM.
